# Expression of *IPT* in Asakura-sanshoo (*Zanthoxylum piperitum* (L.) DC. f. *inerme* Makino) Alters Tree Architecture, Delays Leaf Senescence, and Changes Leaf Essential Oil Composition

**DOI:** 10.1007/s11105-015-0948-9

**Published:** 2015-10-21

**Authors:** Xiao-Fang Zeng, De-Gang Zhao

**Affiliations:** The Key Laboratory of Plant Resources Conservation and Germplasm Innovation in Mountainous Region (Ministry of Education), Institute of Agro-Bioengineering and College of Life Sciences, Guizhou University, Guiyang, 550025 People’s Republic of China

**Keywords:** Asakura-sanshoo, Essential oil, *IPT* gene, Leaf senescence

## Abstract

The *IPT* gene encodes isopentenyl pyrophosphate transferase, a key enzyme in cytokinin biosynthesis. We introduced *IPT* under the control of the CaMV35S promoter into Asakura-sanshoo (*Zanthoxylum piperitum* (L.) DC. f. *inerme* Makino) via stable *Agrobacterium tumefaciens*-mediated transformation. Three of 3-year-old transgenic Asakura-sanshoo lines Y5, Y16, and Y17 were used to evaluate the effects of *IPT* expression on the morphological characteristics, leaf senescence, and essential oil composition. Introduced *IPT* into Asakura-sanshoo stimulated an increase in cytokinin content and a decrease in auxin level. The increase in the cytokinin/auxin ratio affected the tree architecture in 3-year-old transgenic lines. The phenotypes of transgenic lines included reduced stem elongation, decreased leaf surface area, increased branching, and delayed leaf senescence. The expression of *IPT* in Asakura-sanshoo also affected the leaf essential oil composition. The amount of oxygenated sesquiterpenoid compounds in Y5 and Y16 was 21.1 and 15.8 % higher, respectively, than that in wild type (WT). The amount of aromatic compounds in Y5 and Y16 was 2.9 and 24.6 % lower, respectively, than that in WT. These results show that *ipt* expression in Asakura-sanshoo conferred desirable traits, including a dwarf growth habit, delayed senescence, and increased concentrations of some sesquiterpenoid compounds.

**Electronic supplementary material:**

The online version of this article (doi:10.1007/s11105-015-0948-9) contains supplementary material, which is available to authorized users.

## Introduction

Cytokinins regulate many biological processes in plant growth and development. They stimulate cell division, reduce apical dominance and plant elongation, delay plant senescence, prolong the post-harvest shelf-life, and increase production of secondary metabolites (DiCosmo and Misawa 1985; Ha et al. 2012; Hirose et al. 2008; Namdeo 2007; Santoro et al. 2013; Somasundaram 2013; Vankova 2014). The *IPT* gene encodes isopentenyl transferase, which catalyzes the rate-limiting step in de novo cytokinin biosynthesis (Hewelt et al. 1994; McCabe et al. 2001). Genetic modification is one strategy to increase the amount of cytokinin in plants. This has been achieved by introducing the *IPT* gene into the plant genome (Honda et al. 2011; Khodakovskaya et al. 2009). In previous studies, plants overexpressing *IPT* can result in the accumulation of cytokinin, which alters certain morphological and physiological characteristics of the plant, showing delayed leaf senescence, increased abundance of flowers, increased branching, increased secondary metabolite production, restricted root formation, and reduced plant height (Honda et al. 2011; Khodakovskaya et al. 2005; Khodakovskaya et al. 2006; Li et al. 1992).

*Zanthoxylum piperitum* DC., an important deciduous aromatic woody shrub in the Rutaceae, has been used for centuries as an important spice with a fresh flavor. It has also been used in China, Japan, and Korea as a traditional medicine to treat colds, vomiting, diarrhea, and hypotension (Epple et al. 2001; Hwang 2005; Sun et al. 2010; Yanase et al. 2010). Essential oils of *Z. piperitum* show a wide spectrum of biological activities, including strong antimicrobial, insecticidal activities against test organisms, and antioxidant and anticancer activities (Kamsuk et al. 2007; Kim et al. 2003; Lee et al. 1997; Lee and Lim 2008; Negi et al. 2011). Asakura-sanshoo (*Zanthoxylum piperitum* (L.) DC. f. *inerme* Makino) is a thornless variant of *Z. piperitum* (Imaizumi et al. 1993). In Japan, the fruits and leaf of Asakura-sanshoo have been used for centuries as a spice, a vegetable, and a natural drug (Imaizumi 1999).

A dwarf growth habit and branching are two important characters in horticultural plants (Honda et al. 2011). By cultivating dwarf varieties with a high degree of branching, growers can increase plant density and optimize the use of soil, water, and light energy in a limited area (Hansche et al. 1979). Also, it is advantageous to cultivate plants that can be stored for extended periods, that is, those that show delayed senescence of whole plants and excised shoots (Khodakovskaya et al. 2005; Zakizadeh et al. 2013). As a horticultural plant, the main breeding goals of Asakura-sanshoo are to produce plants with high essential oil content, a dwarf growth habit, a high degree of branching, delayed senescence, and long shelf-life of cut shoots. But, as a woody plant, the long generation time of Asakura-sanshoo has been one of the main obstacles to traditional breeding. It is a very slow process to improve and confer dwarfing, branching, and senescence delaying characteristics on woody plants by traditional breeding. In contrast, genetic transformation using genes that affect plant architecture and senescence such as the *IPT* gene provides an alternative approach to develop transgenic plants with leaf senescence delaying and desirable tree shape (Honda et al. 2011; Khodakovskaya et al. 2005; Khodakovskaya et al. 2006). However, to our knowledge, there is no report on *IPT* gene transformation in Asakura-sanshoo.

In this study, with the aim of developing the potential utilization value of dwarf, branching, and senescence delaying transgenic germplasm, the *IPT* gene that comes from *Agrobacterium* (Barry et al. 1984) was introduced into Asakura-sanshoo, and the effects of *IPT* expression on the morphological characteristics, leaf senescence, and essential oil composition of the transgenic lines were evaluated.

## Materials and Methods

### Plant Materials

Stem segments of Asakura-sanshoo cut from growing plants were surface-sterilized with 75 % alcohol for 45 s and then washed with sterile distilled water. Segments were dipped in 0.1 % (*w*/*v*) HgCl_2_ containing Tween 80® for 7 min, washed with sterile water for five times, and dried on sterile paper. The segments were transferred onto axillary shoot-inducing medium Woody Plant medium (WPM) (Lloyd and McCown 1980) supplemented with 1.0 mg/L benzyladenine (BA) and 0.2 mg/L indole-3-butyric acid (IBA) and cultured for 4 weeks at 25 °C under a 16-h light/8-h dark photoperiod. Stem and petiole segments cut from these aseptic axillary shoots were used as explants for transformation.

### Bacterial Strains, Plasmid Vectors, and Plant Genetic Transformation

**Fig. 1:**
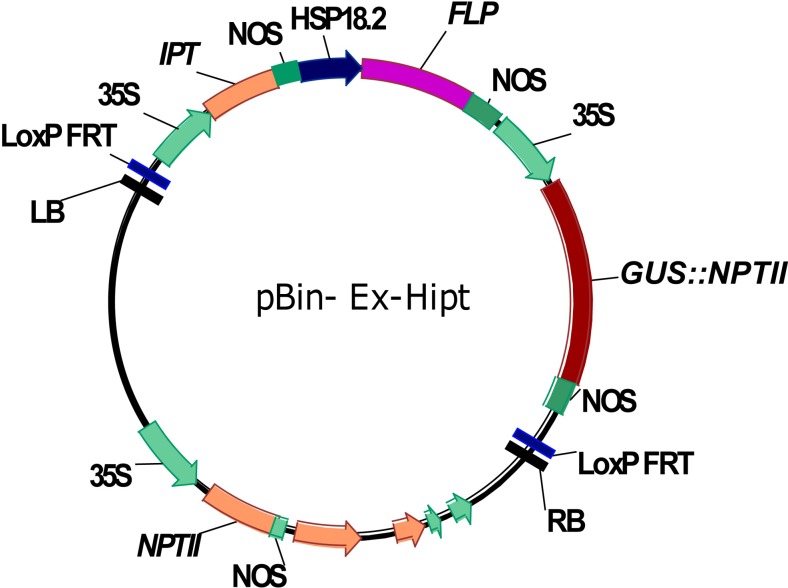
Plant expression vectors pBin-Ex-Hipt. *LB* left border sequence of a T-DNA, *RB* right border sequence of a T-DNA, *35S* the cauliflower mosaic virus 35S (CaMV35S) promoter, the *GUS*-*NPTII* fusion gene and *IPT* gene were all under the control of the 35S promoter, and the *FLP* gene coding for FLP site-specific recombines protein was under the control of HSP 18.2 promoter from *Arabidopsis thaliana*. All the constructs were flanked by two LoxP-FRT fusion sequences as recombination sites in direct orientation

The *Agrobacterium tumefaciens* strain EHA105 containing the pBin-Ex-Hipt plasmid (Fig. 1) was used to transform Asakura-sanshoo. The pBin-Ex-Hipt plasmid contained the fusion uid A gene for β-glucuronidase (*GUS*) and kanamycin-resistant gene (*NPTII*) and *IPT* gene from *Agrobacterium* under the control of the Cauliflower Mosaic Virus 35S (CaMV 35S) promoter and the *FLP* gene encoding the FLP site-specific recombinase protein under the control of a heat shock-inducible promoter HSP 18.2 from *Arabidopsis thaliana*. All the constructs were flanked by two loxP-FRT fusion sites in the sense orientation. A single clone of *A. tumefaciens* strain EHA105 was grown in YEP medium supplemented with 50 mg/L kanamycin and 20 mg/L rifampicin at 28 °C overnight in an incubator with shaking at 200 rpm, until the OD_600_ reached 0.5–0.8. The bacterial cells were resuspended in liquid WPM containing 100 μM acetosyringone (AS) to maintain the OD_600_ at 0.5–0.8. Petiole and stem segments were pre-cultured on WPM supplemented with 0.5 mg/L BA and 0.2 mg/L IBA in the dark at 28 °C for 3 days. Subsequently, the segments were soaked in the bacterial suspension for 10 min. Then, the explants were dried on sterile paper and incubated on co-culture medium (WPM containing 0.5 mg/L BA, 0.2 mg/L IBA, and 100 μM AS) at 28 °C in the dark for 3 days. The explants were transferred to resting medium to recover. After 7 days of resting cultivation, explants were transferred onto selection medium I (WPM supplemented with 0.5 mg/L BA, 0.2 mg/L IBA, 100 mg/L Timentin (ticarcillin sodium and clavulanate potassium), and 30 mg/L kanamycin) for 20 days and then onto selection medium II. For adventitious bud induction, selection medium II (WPM supplemented with 0.5 mg/L BA, 0.2 mg/L IBA, 100 mg/L Timentin, and 50 mg/L kanamycin) was replaced with fresh medium every 3 weeks. The surviving shoots that had elongated to 2–3 cm in length were transferred onto rooting medium for root induction. Well-rooted plantlets were transplanted into pots containing soil and grown in the greenhouse.

### GUS Histochemical Analysis

Histochemical assay of GUS was performed according to Jefferson et al. (1987). Shoots were infiltrated with 1 mM X-GLUC staining solution and incubated at 37 °C overnight. After staining, samples were rinsed with water and fixed in 75 % ethanol.

### Confirmation of Transformation by PCR and Southern Blotting

Total genomic DNA was extracted from fresh leaves of putatively transgenic and wild-type (WT) Asakura-sanshoo plants using a DNAsecure Plant Kit (Tiangen Corp., Beijing, China), according to the manufacturer’s instructions. The PCR amplification conditions were as follows: 94 °C for 5 min; 35 cycles of 94 °C for 30 s, 60 °C for 30 s, and 72 °C for 30 s; and final extension at 72 °C for 7 min. Primers specific for the *IPT* gene were as follows: forward, 5′-GCGCGAAACAGCTATTGGAG-3′; reverse, 5′-TCTGCCG TGATCTGGTTCTG-3′. These primers amplified a 248-bp fragment of the *IPT* gene sequence. The PCR products were examined under ultraviolet light after electrophoresis on a 1 % (*w*/*v*) agarose gel.

Southern blot analysis was according to the protocol of Southern (2006). Briefly, 20 μg genomic DNA and 1 μg control plasmid were digested with *Eco*RI and *Kpn*I (Takara Corp., Dalian, China). The digested genomic DNA and plasmid DNA were separated by 1.0 % agarose gel electrophoresis and transferred onto a Hybond N^+^ membrane. The filter was hybridized at 38 °C with a 325-bp *IPT* specific digoxigenin-labeled probe, which was obtained by PCR amplification from the pBin-Ex-H*ipt* plasmid. The primers specific for the 325-bp *IPT* gene were as follows: forward primer, 5′-TGCTTAACTCT GGCCTTGGC-3′; reverse primer: 5′-ATCGGGTCCAATGCTGTCCTC-3′. This procedure was conducted using a DIG High Prime DNA Labeling and Detection Starter Kit I, according to the manufacturer’s instructions (Roche Applied Sciences, Mannheim, Germany).

### Morphological Analysis of Transgenic Phenotypes and *IPT* Expression Analysis

The transgenic lines and WT were grown in a greenhouse under the same conditions for 3 years. Plant height and leaf size were measured. For the microscopy observation and plant material sectioning, leaf samples were fixed in FAA (formalin/glacial acetic acid/70 % ethanol, 1:1:18) and dehydrated through a graded series of ethanol series. After substitution with xylene, the samples were embedded in paraffin wax and sectioned at 10 μm by using a rotary microtome. Sections were stained with 0.05 % toluidine blue O and observed with a light microscope (Microsystems Wetzlar GmbH; Leica, Germany).

Total RNA was isolated from leaves using a Plant RNA Kit (Omega Bio-Tek, Doraville, GA, USA). Reverse transcription was conducted using a High-Capacity complementary DNA (cDNA) Reverse Transcription Kit (Applied Biosystems, Foster City, CA, USA) with 5 μg RNA as the template. Then, 2 μL reverse cDNA template was used in each 20-μL PCR reaction. Three replicates were analyzed for each sample. The internal reference was 18SRNA, amplified using the following primer pair: forward, 5′-TTAGGCCATG GAGGTTTGAG-3′; reverse, 5′-GAGCTGATGACTCGCGCTTA-3′. Quantitative reverse transcription-PCR (qRT-PCR) analyses were performed using an ABI 7500 Real-Time PCR System and the SYBR® Green PCR Master Mix Kit (Applied Biosystems). Threshold cycles (Ct) for *IPT* expression were standardized to *18S* Ct (−ΔCt). The relative expression level of *IPT* in transgenic plants was determined using the 2^−ΔΔCt^ calculation method (Livak and Schmittgen 2001).

### Extraction and Quantitative Analyses of Cytokinin and Auxin

Leaf tissue of Asakura-sanshoo (0.2 g) was frozen in liquid nitrogen and then ground to a fine powder. Indole acetic acid (IAA), *trans*-zeatin (*trans-*Z), and *trans*-zeatin riboside (*trans-*ZR) were extracted and quantified using the methods described by Zhang et al. (2006) and Wang and Xiao (2009), with some modifications. The extracts in the vials were injected into an HPLC equipped with a YWG-C_18_ stainless-steel column (4.6 mm × 250 mm, 5 μm; Waters, Milford, MA, USA), coupled to an ultraviolet detector (G1314A; Agilent, Palo Alto, CA, USA). The mobile phase was acetonitrile/water (40:60 (*v*/*v*), containing 2.7 mL/L triethylamine, pH 3.0). The sample injection volume was 10 μL, the flow rate was 1.0 mL/min, and the column temperature was 35 °C. The detection wavelengths were 210 nm for IAA and 270 nm for *trans-*Z and *trans-*ZR.

### Senescence of Excised Leaves

To determine the tolerance of plant leaves to dark storage, excised leaves from transgenic lines and WT plants were placed on moist filter paper in 10-cm Petri dish and then stored in the dark at 25 °C. The chlorophyll concentration was assayed using the method of Inskeep and Bloom (1985) before the start of each experiment and after 10 and 15 days of the dark storage treatment.

### Leaf Essential Oil Composition

The leaf essential oils from WT and the transgenic lines Y5 and Y16 were subjected to direct headspace sampling using solid-phase micro extraction (SPME) using a Supelco 2-cm 50/30 μm SPME fiber and analyzed by gas chromatography/mass spectrometry (GC/MS). The GC/MS system (Agilent 5975) consisted of a gas chromatograph (Agilent 6890) with a mass selective detector (Agilent 5973) and a ChemStation data system. The GC column was a Zebron ZB-5 ms fused silica capillary column (30 m × 0.25 mm × 0.25 μm, 5 % phenyl–95 % dimethylpolysiloxane). The carrier gas was helium (high purity, 99.999 %) with a column head pressure of 7.62 psi and a flow rate of 1.0 mL/min. The GC oven temperature program was as follows: 40 °C initial temperature, hold for 2 min, and increase at 4 °C/min to 150 °C. The mass spectrometer was operated at 70 eV. The chemical constituents of the oils were identified by comparing their mass spectral patterns and retention indices with those in the Wiley 225 GC/MS library, as described by Adams (2007).

## Results

### Establishment and Molecular Analysis of Transgenic Plants

**Fig. 2:**
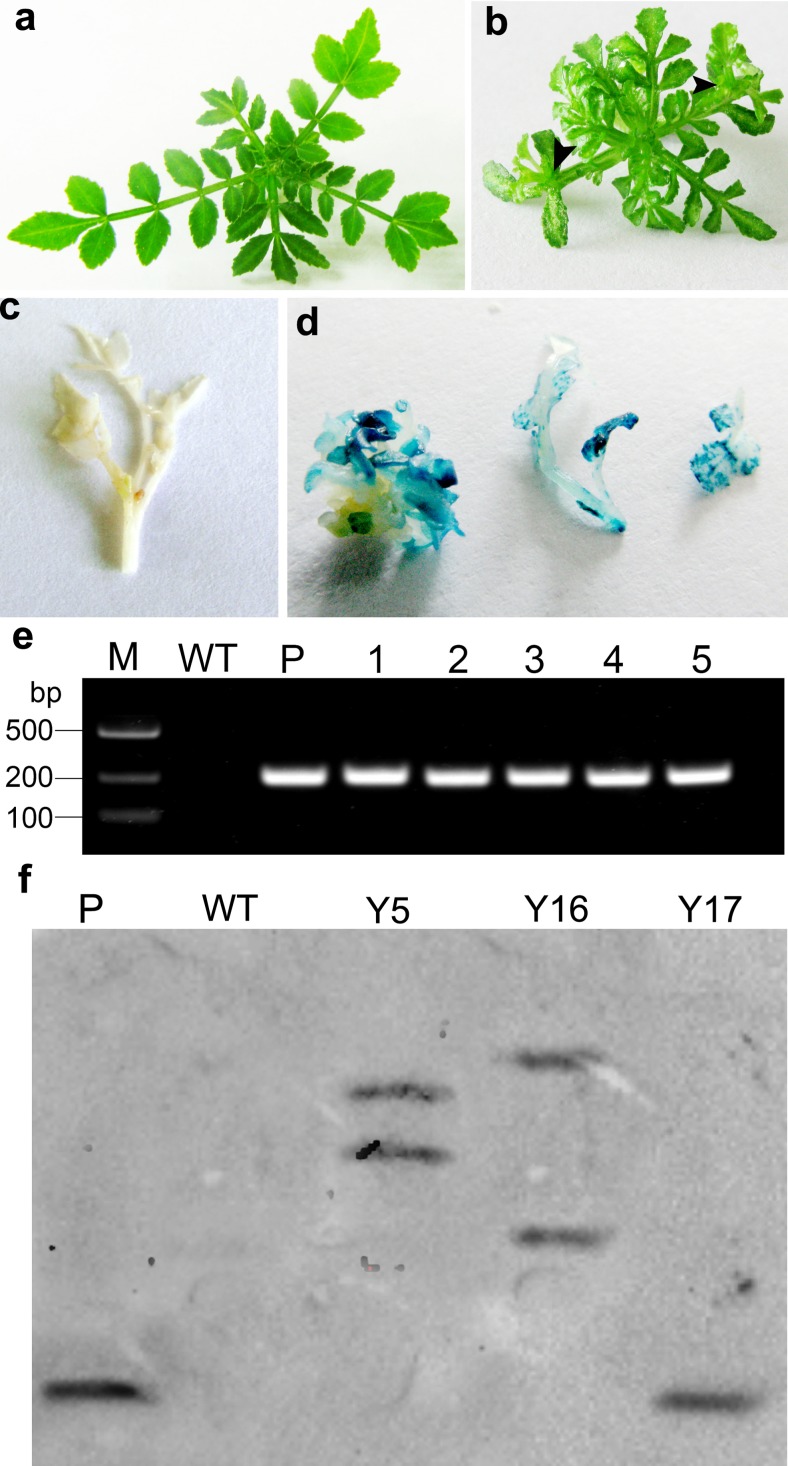
Kanamycin-resistant shoots, GUS histochemical detection, and PCR analysis. **a** WT shoots. **b** Kanamycin-resistant shoots. *Black arrows* show epiphyllous shoots. **c** WT shoots. **d** GUS-positive shoots and leaves. **e** PCR analysis of transgenic Asakura-sanshoo plants. *M* DL2000 DNA marker, *WT* DNA from non-transgenic plant, *P* plasmid, *1–5* transgenic lines. **f** Southern blotting analysis. *P* pBin-Ex-Hipt plasmid; *WT* genomic DNA from non-transgenic plant; *Y1*, *Y17*, *Y5*, and *Y16* genomic DNA from transgenic lines. Genomic DNA from all lines and plasmid DNA were digested with *Eco*RI and *Kpn*I

The *IPT* gene under the control of 35S promoter was introduced to Asakura-sanshoo plants, and more than 1000 kanamycin-resistant shoots were obtained. Most of the resistant shoots showed typical cytokinin-overproducing responses such as restrained apical dominance, increased branching, and emergent epiphyllous shoots (Fig. 2a, b). It was difficult to induce root formation from these shoots (37.5 % in transgenic shoots, while 86.1 % in WT). Addition of 2.0 mg/L IBA reduced the inhibition of root formation, and the rooting frequency in transgenic shoots increased to 75.0 % (Table 1). At last, only 35 well-rooted transgenic shoots were obtained. These plantlets were tested for GUS activity and then transferred into pots and grown in a greenhouse. To verify the transgenic plants, genomic DNA was isolated from the kanamycin-resistant, GUS-positive plantlets, and PCR analysis was carried out using specific primers for *IPT*. As expected, a 248-bp *IPT* specific fragment was amplified from the positive control as well as from all five analyzed plants. This fragment was not detected in WT plants (Fig. 2e). A Southern blot analysis revealed that only one copy of the *IPT* gene was present in the genome of line Y17, and more than one copy was present in the genomes of lines Y5 and Y16. No hybridization signal was detected in WT plants. These results indicate that the genome of WT Asakura-sanshoo does not contain a sequence homologous to that of the *IPT* gene (Fig. 2f).

**Table 1:** Effects of *IPT* expression on root formation from shoots

Medium	Rooting frequency (%)	
	WT	Transgenic shoots
1/2WPM	86.11 ± 2.98a	37.5 ± 1.13b
1/2WPM + IBA 2.0 mg/L	55.0 ± 1.16b	75.0 ± 2.86a

Values are mean ± SD (*n* = 3). Different lowercase letters represent significant difference (Tukey’s test for LSD, *P* ≤ 0.01)

### Morphological Phenotypes and *IPT* Expression in Transgenic Plants

**Fig. 3:**
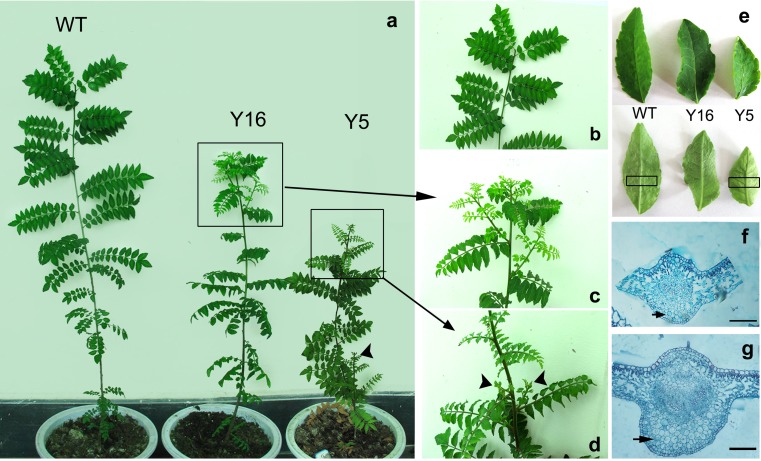
Phenotypes of 3-year-old transgenic Asakura-sanshoo expressing *IPT*. **a** WT and transgenic plants (*Y16* and *Y5*) grown in soil. *Arrows* show lateral shoots. **b–d** Dormancy of lateral buds of WT, and broken dormancy of lateral buds in transgenic lines Y5 and Y16. *Arrows* showed the lateral shoots. **e** Leaves from transgenic plants (*Y5* and *Y16*) and non-transgenic plants (*WT*); *arrows* showed the coarser leaf midribs in Y5 and Y16. **f** Cross sections of leaf from WT, taken from **e** area highlighted in *black* (enlarged ×200); the *black arrow* indicates the parenchyma cells. **g** Cross section of leaf from Y5, taken from **e** area highlighted in *black* (enlarged ×200); the *black arrow* indicates the parenchyma cells, *bar* = 200 μm

**Fig. 4:**
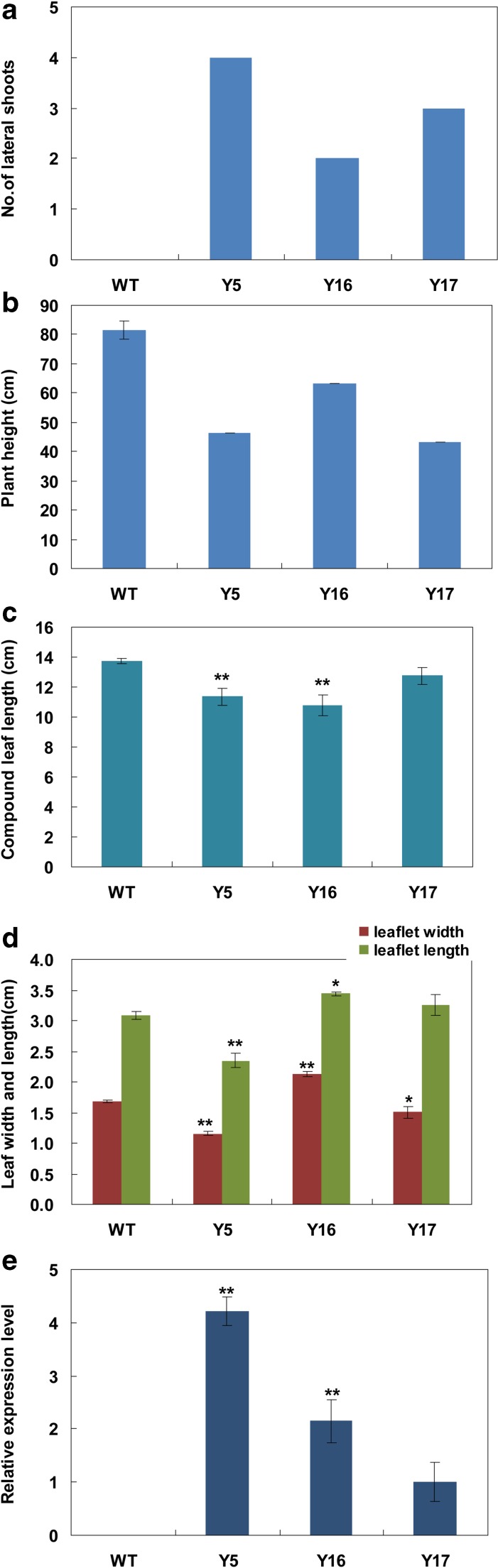
Morphological changes in 3-year-old transgenic plants expressing *IPT* and *IPT* gene expression levels in leaves of transgenic plants. **a** Number of lateral branches. The data of WT were statistic of five WT plants. **b** Plant height. The data of WT were statistic of five WT plants. **c** Compound leaf length. Significant difference at *P ≤ 0.05 (Tukey’s test for LSD) and **P ≤ 0.01 (Tukey’s test for LSD) (*n* = 3). **d** Leaf size. Significant difference at *P ≤ 0.05 (Tukey’s test for LSD) and **P ≤ 0.01 (Tukey’s test for LSD) (*n* = 3). **e** Expression levels of *ipt. Error bars* represent SD. Significance at *P ≤ 0.05 and **P ≤ 0.01 (*n* = 3)

Transgenic lines of Asakura-sanshoo expressing *IPT* showed a range of phenotypes related to cytokinin overproduction. Three-year-old transgenic lines showed various physiological and morphological abnormalities, including weak apical dominance, dwarfism, and smaller leaves (Figs. 3a–d and 4a, b). Transgenic Asakura-sanshoo plants expressing *IPT* also showed reduced dormancy of auxiliary buds. Auxiliary buds outgrew from nodes on the main stem in transgenic lines, especially Y5. The auxiliary buds originated from basal nodes in Y5, but no auxiliary buds grew from nodes of 3-year-old WT plants in the same conditions (Figs. 3a and 4a). The transgenic lines showed reduced stem elongation, resulting in a dwarf trait (Figs. 3a and 4b). Compared with WT plants, transgenic lines had smaller compound leaves and shorter leaflets (Figs. 3e and 4c–d). Cross sections of Y5 leaves showed that the parenchyma cells were more abundant and larger than those in WT leaves (Fig. 3f–g). A qRT-PCR analysis showed that the level of *IPT* expression in Y5 and Y16 was 4.20-fold and 2.15-fold higher, respectively, than that in Y17 (Fig. 4e). This result indicated that the high *IPT* expression level in Y5 caused its extreme morphological differences from WT.

### Endogenous Contents of Cytokinins and Auxin in *IPT* Transgenic Asakura-Sanshoo

There were differences between transgenic lines and WT in the concentrations of bioactive cytokinins, cytokinin phosphates, and IAA (Table 2). The cytokinin levels were higher in leaves of all *IPT*-transformed lines than that in WT plants. The contents of *trans*-Z in leaves of Y5, Y16, and Y17 plants were 4.14-, 2.72-, and 1.86-fold higher, respectively, than that in WT. Compared with that in WT plants, the contents of the active cytokinin precursor *trans-*ZR were 11.96-, 10.48-, and 8.90-fold higher in Y5, Y16, and Y17 plants, respectively. However, the IAA contents were markedly lower in transgenic lines than in WT plants. The IAA levels in transgenic lines Y5, Y16, and Y17 were reduced to 5.4, 5.7, and 7.9 % of that in WT plants, respectively. The *IPT* expression level was directly correlated with the cytokinin (*trans-*Z + *trans-*ZR)/IAA ratio (Table 2). The higher cytokinin/IAA ratio in Y5 that had the highest *IPT* expressing level caused the most severe phenotype among the three transgenic lines.

**Table 2:** Endogenous cytokinin and auxin contents in the transgenic and wild-type Asakura-sanshoo plants

Lines	IAA (ng/g FW)	Z (ng/g FW)	ZR (ng/g FW)
WT	115.55 ± 6.80a	1.25 ± 0.14d	13.11 ± 0.70d
Y5	6.23 ± 0.91b	5.18 ± 0.24a	156.79 ± 2.33a
Y16	6.54 ± 0.47b	3.40 ± 0.13b	137.38 ± 3.69b
Y17	9.13 ± 2.58b	2.33 ± 0.30c	116.66 ± 13.8c

Data presented are mean ± SD, *n* = 3. Different lowercase letters represent significant difference by Tukey’s test for LSD at *P* ≤ 0.05

*Z* trans-zeatin, *ZR* trans-zeatin riboside, *IAA* indoleacetic acid

### Delayed Leaf Senescence of P_*35S*_*-IPT* Transgenic Asakura-Sanshoo Lines

**Fig. 5:**
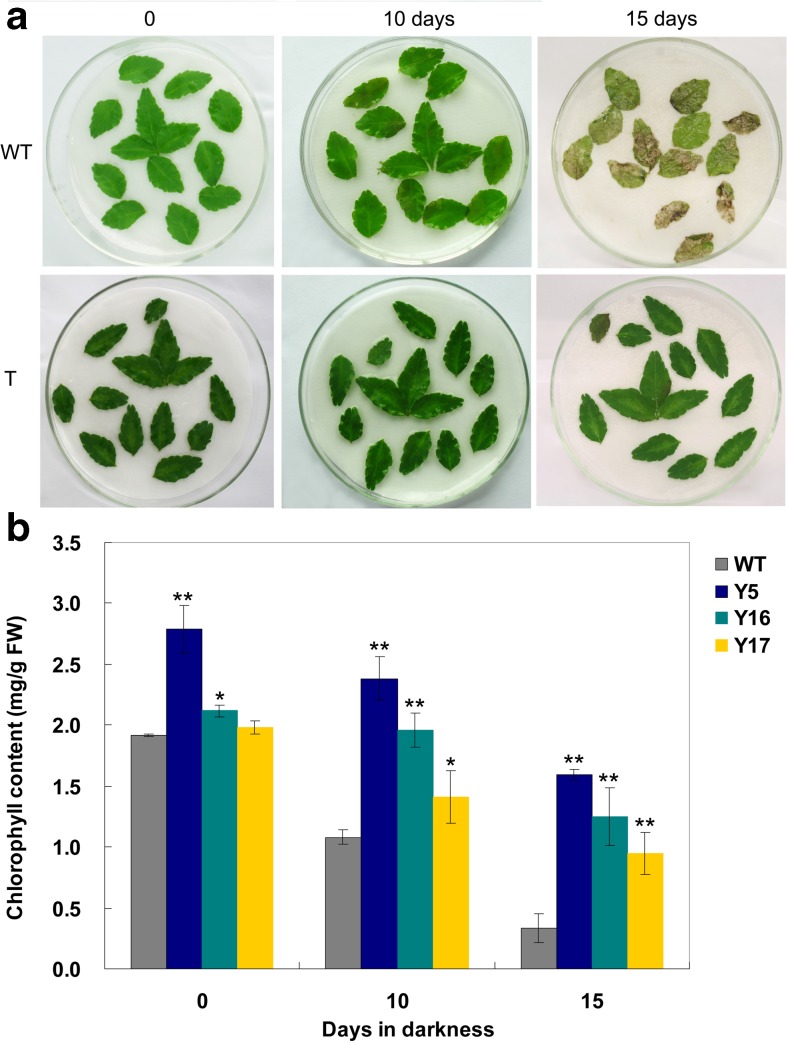
Delayed senescence of excised leaves of 3-year-old transgenic Asakura-sanshoo expressing *IPT*. **a** Excised leaves of Y17 (*T*) and WT plants at 0, 10, and 15 days of storage in darkness. **b** Chlorophyll content of leaves of transgenic and WT Asakura-sanshoo plants. *Error bars* represent SD. Significance at *P ≤ 0.05 and **P ≤ 0.01 (*n* = 3)

Leaves from the transgenic lines Y5, Y16, and Y17 and WT were kept in darkness in vitro to observe their senescence responses. Compared with WT, transgenic lines showed delayed leaf senescence (Fig. 5). The chlorophyll content was lower in WT leaves than that in transgenic leaves under normal greenhouse conditions. The increase in endogenous cytokinin caused 3.1–45.3 % increases in chlorophyll accumulation in transgenic lines. The level of *IPT* expression was directly correlated with cytokinin content and delayed leaf senescence. The total chlorophyll contents decreased in transgenic lines and WT after 10 and 15 days in darkness. However, the relative decrease in chlorophyll content differed between WT and transgenic lines; after 10 days in darkness, the leaves of WT showed symptoms of chlorophyll loss and turned yellow and thinned, and the leaves of transgenic lines presented little or no visible symptoms of chlorophyll loss. There were a 43.7 % loss of chlorophyll in WT, but only 7.6, 14.5, and 28.9 % chlorophyll losses in lines Y5, Y16, and Y17, respectively. After 15 days in darkness, the chlorophyll content had decreased by 82.6 % in WT leaves, but only to 47.9–58.9 % of the initial level in transgenic lines.

### Leaf Essential Oil Composition

Essential oils extracted from the leaves of WT and transgenic plants were analyzed by SPME-GC/MS. In WT leaves, 41 constituents represented 98.6 % of total essential oils; in Y5, 36 constituents represented 95.4 % of total essential oils, and in Y16, 40 constituents represented 96.0 % of total essential oils (Table 3, Table S1). The chemical composition of essential oils from leaves of transgenic and non-transgenic Asakura-sanshoo is shown in Table S1.

**Table 3:** Chemical composition of essential oils from leaves of transgenic and wild-type Asakura-sanshoo

Constituent	Area (%)^a^		
	WT	Y5	Y16
Aromatic compounds	41.27c	38.38b	16.73a
Monoterpenes	20.93c	6.05a	16.98b
Oxygenated monoterpenes	11.79b	4.86a	17.35c
Sesquiterpenoids	2.56a	2.81a	6.83b
Oxygenated sesquiterpenoids	20.38a	41.51b	36.16c
Sterides, fatty hydrocarbon, fatty acids, and simple oxygenated compounds	1.67	1.78	1.90

Different lowercase letters represent significant difference (Tukey’s test for LSD, *P* ≤ 0.01; *n* = 3)

^a^Indicated the oil content and composition are in percentages (%)

The essential oils extracted from WT leaves consisted of aromatic compounds (41.3 %), monoterpene hydrocarbons (20.9 %), oxygenated sesquiterpenoids (20.4 %), and oxygenated monoterpenoids (11.8 %), with smaller amounts of sequiterpene hydrocarbons (2.6 %), sterides, fatty hydrocarbons, fatty acids, and simple oxygenated compounds (1.7 %) (Table 3). The main components of leaf essential oils differed between transgenic lines and WT. Compared with that in WT, the amount of oxygenated sesquiterpenoid compounds in Y5 and Y16 was 21.1 and 15.8 % higher, respectively. There were small increases in sesquiterpenoid contents in Y5 and Y16 (0.2 and 4.3 %, respectively), but these increases were insignificant. Compared with WT, Y5 and Y16 showed 2.89 and 24.6 % decreases, respectively, in the amount of aromatic compounds. The amount of monoterpenoids was 14.9 and 4.0 % lower in Y5 and Y16, respectively, than that in WT (Table S1). The major components of essential oils from wild-type leaves were methyl cinnamate (39.6 %), farnesol (20.0 %), α-pinene (13.1 %), citronellal (8.2 %), and α-terpinene (2.8 %). The major components of essential oils from leaves of line Y5 were methyl cinnamate (35.3 %), d-nerolidol (34.6 %), farnesyl acetate (5.3 %), α-pinene (2.9 %), citronellal (2.5 %), and β-caryophyllene (2.2 %). The major components of essential oils from leaves of transgenic line Y16 were farnesol (33.4 %), methyl cinnamate (15.6 %), α-pinene (11.4 %), citronellol (6.3 %), and linalool (5.9 %).

## Discussion

Cytokinins and auxins are important regulators of plant growth and development. The cytokinin/auxin ratio is an important factor in shoot branching (Ongaro and Leyser 2008; Shimizu-Sato et al. 2009). In horticulture, people often prune or exogenously applied cytokinins to improve the cytokinin/auxin ratio so as to encourage growth of branches. Expression of *IPT* in transgenic plants can lead to extreme cytokinin overproduction, resulting in lower levels of IAA, and then leading to typical cytokinin overproduction symptoms including weaker apical dominance, reduced root initiation and growth, smaller leaves, and reduced plant height (Hewelt et al. 1994; Li et al. 1992). This usage of *IPT* gene has also been used to alter plant architecture by increasing branching and/or reducing stem elongation in several woody horticultural plant species (Honda et al. 2011; Khodakovskaya et al. 2009). In this study, transgenic Asakura-sanshoo plants expressing the *IPT* gene showed elevated endogenous cytokinin levels. The transcript level of *IPT* in transgenic Asakura-sanshoo plants was positively correlated with cytokinin content and negatively correlated with auxin content, as observed in other plant species. The cytokinin-overproducing transgenic lines analyzed in this study showed lower IAA concentrations than that in WT. The elevated cytokinin/IAA ratio caused a range of morphological and physiological changes in transgenic Asakura-sanshoo plants. Compared with WT plants, the transgenic lines showed increased branching, reduced apical dominance, reduced leaf surface area, and shorter stems (Figs. 2 and 3).

Leaf senescence is a programmed process representing the final phase of leaf development (Sykorova et al. 2008). Leaf senescence is related to a decrease in the cytokinin content in leaves (Gan and Amasino 1995; McCabe et al. 2001; Zakizadeh et al. 2013). Expression of the *IPT* gene has been shown to increase the level of endogenous cytokinin and delay leaf senescence in various plant species (Ainley et al. 1993; Khodakovskaya et al. 2009; Klee et al. 1987). Introducing the *IPT* gene into broccoli and lettuce delayed post-harvest leaf senescence and extended their shelf-life (Chen et al. 2001; McCabe et al. 2001). In this study, transgenic Asakura-sanshoo plants expressing the *IPT* gene showed increased chlorophyll content and delayed leaf senescence, compared with WT plants. After 10 days in darkness, leaves of WT plants turn yellow and thinned, and leaves from transgenic plants remained dark green. At the end of dark treatment of the in vitro culture experiment (15 days), most of the leaves of both WT and transgenic plants had senesced. In WT plants, almost all the leaves turned yellow, whereas senesced leaves from the *IPT* transgenic plants remained green, and a few yellow leaves were observed.

In several studies, the senescence-specific gene from *A. thaliana* leaf *SAG12* promoter was used to control the expression of *IPT*, so that to obtain the senescence delayed transgenic plants without phenotypic abnormalities (Gan and Amasino 1995; Zakizadeh et al. 2013). The expression of *ipt* gene controlled by *CaMV35S* leads to extreme overproduction of cytokinins in transgenic plants. By using 35S promoter, we can obtain not only senescence delayed but also with branching increased and dwarf transgenic plants. And the same usage of P_*35S*_*-IPT* was also reported in woody tree kiwifruit (Honda et al. 2011) and poplar (von Schwartzenberg et al. 1994). Asakura-sanshoo is a thornless variant of *Z. piperitum.* Because of its lack of thorns, it is more suitable than thorny cultivars for harvesting of young sprouts and fruits. The transgenic lines with a dwarf phenotype (Figs. 2a and 3b), increased branching (Figs. 2a and 3a), and delayed senescence (Fig. 4) illustrate that the P_*35S*_*-IPT* construct has potential uses in improving fruit and young sprout production, and prolonging the picking and storage periods of young sprouts also exists in other plant species. Meanwhile, the character of dwarf and branching in transgenic plants could drastically reduce pruning requirements, so as to save cost.

Currently, many reports showed the relationship between the changes of cytokinin content and the production of secondary metabolites (Drawert et al. 1984; Santoro et al. 2013). Their results suggested that different concentrations of cytokinins could affect secondary metabolite production. Changes in the contents of endogenous cytokinin as a result of *IPT* gene expression in transgenic plants and the effects of *IPT* on the production of secondary metabolites have also been described in some reports. Smigocki et al. (1993) showed that introducing the *IPT* gene into tobacco increased the activity of the secondary metabolic pathway, resulting in increased insect resistance in transgenic plants. Geng et al. (2001) suggested that an increase in endogenous cytokinin levels could stimulate an increase in metabolic activity, thereby increasing the content of artemisinin an effective antimalarial drug against *Plasmodium falciparum* in transgenic *Arabidopsis thaliana* plants expressing the *IPT* gene. Imaizumi et al. (1993) reported that branching was correlated with the levels of aromatic secondary metabolites in Asakura-sanshoo. Their results showed that shoot-tip cultured lines with more branches contained higher contents of essential oil. In our study, transgenic Asakura-sanshoo plants expressing the *IPT* gene showed changes in leaf essential oil composition, compared with that in WT. Transgenic plants contained higher contents of terpenoids, especially oxygenated sesquiterpenoids. The levels of the oxygenated sesquiterpenoids d-nerolidol and farnesol were significantly higher in Y5 and Y16 than those in WT. Terpenoids play important roles in plant-environment interactions and plant-plant communication and show numerous ecological functions in the plant, antioxidant activity, and spicy aroma (Bakkali et al. 2008; Lange and Ahkami 2013). The volatile sesquiterpenoids and oxygenated sesquiterpenoids such as β-caryophyllene, farnesol, farnesyl acetate, and d-nerolidol with special fragrance and anti-inflammatory, antibiotic, antioxidant, and anticarcinogenic biological activities have traditionally been used in cosmetic, perfumes, and antibiotic, anesthetic, and anti-inflammatory agents (Burke et al. 2001; Lee et al. 2007; Legault and Pichette 2007). The increase of contents in these chemical compositions in transgenic Asakura-sanshoo plants might improve the potential medicinal and cosmetic uses and values in this plant.

Sakakibara et al. (2006) suggested that cytokinin could regulate the expression of genes involved in secondary metabolic pathways in *A. thaliana* through repressing nitrate transporters. To our knowledge, the genome sequence of Asakura-sanshoo has not been fully determined yet. Further research is required to explore the relationship between *IPT* gene expression and essential oil composition in transgenic plants.

## Electronic Supplementary Material

Below is the link to the electronic supplementary material.

ESM 1 Table S1 Chemical composition of essential oils from leaves of transgenic lines Y5 and Y16 and wild-type Asakura-sanshoo. (DOC 153 kb)
