# Supplementary material for: Expression of IPT in Asakura-sanshoo (Zanthoxylum piperitum (L.) DC. f. inerme Makino) Alters Tree Architecture, Delays Leaf Senescence, and Changes Leaf Essential Oil Composition
Source: Plant Mol Biol Report. 2015 Oct 21;34:649–58. doi: 10.1007/s11105-015-0948-9 (PMC4848336; doi:10.1007/s11105-015-0948-9)
Supplement: Supplementary file 1 — Table S1 Chemical composition of essential oils from leaves of transgenic lines Y5 and Y16 and wild-type Asakura-sanshoo. (DOC 153 kb) [file 11105_2015_948_MOESM1_ESM.doc]

**Table S1** Chemical composition of essential oils from leaves of transgenic lines Y5 and Y16 and wild-type Asakura-sanshoo.

| Peak | Constituent | Area^a^ (%) | | |
| --- | --- | --- | --- | --- |
|  |  | WT | Y5 | Y16 |
| **Monoterpenes** | |  |  |  |
| 1 | α-Pinene | 13.09 | 2.87 | 11.40 |
| 2 | Camphene | 0.10 | - | 0.08 |
| 3 | β-Phellandrene | 0.53 | 1.35 | - |
| 4 | Sabinene | - | - | 0.10 |
| 5 | 1-β-Pinene | - | - | 0.11 |
| 6 | 2-β-Pinene | 0.99 | 0.38 | 1.11 |
| 7 | γ-Terpinene | 2.24 | - | 3.97 |
| 8 | (E)- β-Ocimene | - | - | 0.13 |
| 9 | α- Terpinene | 2.84 | 0.87 | 0.08 |
| 10 | (+)-M-Mentha-1, 8-diene | - | 0.28 | - |
| 11 | α-Phellandrene | 1.08 | - | - |
| 12 | α-Thujene | 0.07 | - | - |
| 13 | Cyclohexane,1-methyl-4-(1-methylethenylidene-, *trans*) | - | 0.31 | - |
|  |  |  |  |  |
| **Oxygenated monoterpenes** | |  |  |  |
| 14 | 1, 8-Cineole | 2.04 | 0.21 | 0.59 |
| 15 | *cis*-Sabinene hydrate | 0.10 | - | - |
| 16 | Linalool | - | - | 5.92 |
| 17 | Citronellal | 8.16 | 2.53 | 3.45 |
| 18 | α-Terpinenyl acetate | - | - | 0.34 |
| 19 | (E)-Carveol | - | - | 0.06 |
| 20 | Citronellyl acetate | - | 1.58 | - |
| 21 | Citronellol | 0.52 | - | 6.32 |
| 22 | Piperitone | 0.27 | 0.23 | 0.34 |
| 23 | α-Citral | 0.14 | - | 0.07 |
| 24 | Citronellyl acetate | 0.32 | - | 0.09 |
| 25 | β-Ionone | 0.26 | 0.31 | 0.16 |
|  |  |  |  |  |
| **Sesquiterpenoids** | |  |  |  |
| 26 | β-caryophyllene | 1.61 | 2.20 | 5.06 |
| 27 | α-Humulene | 0.34 | - | 1.18 |
| 28 | Isocaryophyllene | - | - | 0.11 |
| 29 | *trans*-β-Farnesene | 0.06 | 0.11 | - |
| 30 | E, E-α-Farnesene | 0.50 | 0.24 | 0.20 |
| 31 | γ-Muurolene | - | - | 0.10 |
| 32 | Δ-Cadinene | 0.05 | 0.27 | 0.18 |
|  |  |  |  |  |
| **Oxygenated sesquiterpenoids** | |  |  |  |
| 33 | d-Nerolidol | - | 34.63 | - |
| 34 | Farnesol | 19.96 | 1.62 | 33.37 |
| 35 | Caryophyllene oxide | - | - | 0.28 |
| 36 | Farnesyl acetate | 0.42 | 5.26 | 2.52 |
|  |  |  |  |  |
| **Aromatic compounds** | |  |  |  |
| 37 | Benzoic acid, methyl ester | 0.30 | - | - |
| 38 | Benzoic acid, ethyl ester | 0.15 | - | - |
| 39 | Naphthalene | - | - | 0.08 |
| 40 | methyl salicylate | - | - | 0.16 |
| 41 | Benzoic acid, 2-hydroxy-, methyl ester | 0.26 | 0.07 | - |
| 42 | 1H-Indole | 0.55 | - | - |
| 43 | (z)-3-Phenyl-2-propenoic acid, methyl ester | 0.08 | - | - |
| 44 | Bis (2-methoxyethyl) phthalate | - | - | 0.23 |
| 45 | 1, 2-Benzenedicarboxylic acid, dipropyl ester | 0.07 | - | - |
| 46 | 1, 2-Benzenedicarboxylic acid, bis (2-methoxyethyl) ester | - | 0.55 | - |
| 47 | 4, 4’-Bis (4-ethoxybenzyl) diselenide | 0.05 | - | - |
| 48 | Phenol, 5-methyl-2-(1-methylethyl)- | - | 0.56 | - |
| 49 | Butyl phthalate | - | - | 0.30 |
| 50 | 1-ethoxy-4-Ethylbenzene | - | 0.03 | - |
| 51 | Methyl cinnamate | 39.64 | 35.34 | 15.63 |
| 52 | 1, 2-benzenedicarboxylic acid, dimethyl ester | - | 0.68 | - |
| 53 | Ethyl cinnamate | 0.09 | - | - |
| 54 | 2-Proenoic acid, 3-henyl-, ethyl ester | - | 0.40 | - |
| 55 | Ionone | - | - | 0.33 |
| 56 | Phenol, 2, 6 -bis (1, 1- dimethylethyl) -4-methyl | 0.09 | - | - |
| 57 | Butyl hydroxy toluene | - | 0.75 | - |
|  |  |  |  |  |
| **Sterides, fatty hydrocarbon, fatty acids, and simple oxygenated  compounds** | |  |  |  |
| 58 | 2-Butyn-1-ol | 0.59 | - | - |
| 59 | (E) 2-Hexenal | - | 0.35 | 0.10 |
| 60 | z-3-hexenol | - | 0.35 | - |
| 61 | 2-Cyclopenten-1-one | - | 0.05 | - |
| 62 | (E, E)-2,4-hexadienal | - | 0.07 | 0.06 |
| 63 | Hexanoic acid, ethyl ester | 0.07 |  | - |
| 64 | (z)-3-Hexenyl acetate | - | - | 0.59 |
| 65 | z-Undecanone | - | 0.03 | 0.57 |
| 66 | 2, 4-Dimethylcyclohex-4-ene-1,3-dione | 0.09 | - | - |
| 67 | Ethyl, 3-(2-fruyl)-propanoate | - | - | 0.18 |
| 68 | Hexen-1-ol, acetate,(z)- | 0.49 | - | - |
| 69 | Acetamide | - | 0.06 | - |
| 70 | Androst-16-en-3-ol, (3α,5α)- | 0.05 | - | - |
| 71 | (2, 4, 4, 4, 16, 16-D6)-3-2, 17, β-Dihydroxy-5, β-androstane | - | 0.07 | - |
| 72 | 1, 3-Pentadiene, 2, 3-dimethyl | 0.23 | - | - |
| 73 | 2-methyl-1, 3-pentadiene | - | - | 0.14 |
| 74 | 1-Nonadecene | 0.09 | - | - |
| 75 | Cycloentane, 1, 3-bis (methylene-) | - | 0.12 | - |
| 76 | 1, 3-Pentadiene, 2-methyl, trans | - | 0.46 | - |
| 77 | Tetracosanoic acid, methyl ester | 0.06 | - | - |
| 78 | Hexadecanoic acid, methyl ester | - | 0.22 | - |
| 79 | Ethyl, 3-(2-Furyl)-propanoate | - | - | 0.18 |
| 80 | Ethyl caproate | - | - | 0.08 |

^a^ values were mean (*n*= 3) and Oil content and composition are in percentages (%)
